# The Interactive Effects of Ammonia and Microcystin on Life-History Traits of the Cladoceran *Daphnia magna*: Synergistic or Antagonistic?

**DOI:** 10.1371/journal.pone.0032285

**Published:** 2012-03-05

**Authors:** Zhou Yang, Kai Lü, Yafen Chen, David J. S. Montagnes

**Affiliations:** 1 Jiangsu Province Key Laboratory for Biodiversity and Biotechnology, School of Biological Sciences, Nanjing Normal University, Nanjing, China; 2 State Key Laboratory for Lake Science and Environment, Nanjing Institute of Geography and Limnology, the Chinese Academy of Sciences, Nanjing, China; 3 Institute of Integrative Biology, Biosciences Building, University of Liverpool, Liverpool, United Kingdom; Institute of Marine Research, Norway

## Abstract

The occurrence of *Microcystis* blooms is a worldwide concern that has caused numerous adverse effects on water quality and lake ecology. Elevated ammonia and microcystin concentrations co-occur during the degradation of *Microcystis* blooms and are toxic to aquatic organisms; we studied the relative and combined effects of these on the life history of the model organism *Daphnia magna*. Ammonia and microcystin-LR treatments were: 0, 0.366, 0.581 mg L^−1^ and 0, 10, 30, 100 µg L^−1^, respectively. Experiments followed a fully factorial design. Incubations were 14 d and recorded the following life-history traits: number of moults, time to first batch of eggs, time to first clutch, size at first batch of eggs, size at first clutch, number of clutches per female, number of offspring per clutch, and total offspring per female. Both ammonia and microcystin were detrimental to most life-history traits. Interactive effects of the toxins occurred for five traits: the time to first batch of eggs appearing in the brood pouch, time to first clutch, size at first clutch, number of clutches, and total offspring per female. The interactive effects of ammonia and microcystin appeared to be synergistic on some parameters (e.g., time to first eggs) and antagonistic on others (e.g., total offspring per female). In conclusion, the released toxins during the degradation of *Microcystis* blooms would result, according to our data, in substantially negative effect on *D. magna*.

## Introduction

Cyanobacterial blooms occur worldwide in eutrophic lakes and reservoirs [1]. Such blooms are of great ecological concern, as many genera of bloom-forming cyanobaceria produce cyanotoxins [1]–[3]. Furthermore, there is an expectation that such blooms are likely to increase in prevalence and magnitude in the future, especially with climate change [4]. Microcystins, which are cyclic heptapeptides and a main group of the cyanotoxins, are mainly retained within the producer-cells during cyanobacterial bloom development [5]. Upon ingestion, microcystins are actively absorbed by fish, birds, and mammals, and have adverse effects on these aquatic organisms [6]–[8].

To date, concentrations of dissolved microcystins have been measured to be generally <10 µg L^−1^ [9], [10]. However, recent studies have revealed much higher levels. Microcystin levels can be as high as 10 µg L^−1^ in localized regions of eutrophic lakes: e.g. this has been observed in Lake Taihu, which is typical of many lakes in the Asian subcontinent [10], and especially during the water crisis of Lake Taihu in 2007, microcystin concentrations reached >15 µg L^−1^ [11]. Furthermore, in a few cases, microcystin levels be >1800 µg L^−1^ when toxins are released into water after lysis of cyanobacterial cells, during the collapse of heavy blooms [12], [13]. These observations of high levels, which may become more common as cyanobacterial blooms increase in frequency and become more pronounced [4], stimulated us to examine a range of concentrations that include extremely high concentrations. The released toxins can then come into contact with a wide range of aquatic organisms and have deleterious effects on them [3], [5], [7], [8], [14]–[16]. Specially, microcystin inhibits feeding, reduces growth, and increases mortality in cladocerans [17]–[20], and in particular, the variant microcystin-LR exerts strong toxic effects on *Daphnia* [18], [21]–[26].

In general, due to the degradation of organic martial formed by phytoplankton blooms, ammonia can be produced [27]. Such ammonia levels normally are very low, except under conditions of rapid decomposition of organic matter. Thus, like microcystin, during the degradation of heavy *Microcystis* blooms, elevated concentrations of ammonia occur and can reach surprisingly high levels: e.g., 0.2–3.4 mg L^−1^ in areas of Lake Taihu at specific times [28] and ∼4 mg L^−1^ with localized peaks of ∼12 mg L^−1^ during the water crisis in the same lake [11]. Also, total ammonia in localized regions of other freshwaters, e.g. the Mississippi, can be on the order of is about 0.07–4 mg L^−1^ [29]. It is well accepted that high ammonia levels are deleterious to cladocerans and can result in mortality, inhibited growth, and reduced reproductive performance [30]–[35]; here we, therefore, considered medium and very high levels of this toxin, following the same arguments made for microcystin, above. Finally, and importantly, it might also be expected that when ammonia and microcystin are simultaneously released, and persist for days in the region of blooms [9], that they have combined impacts on aquatic organisms, especially cladocerans; however, there is little, if any, information available on their combined effects.

It is clearly important to examine the influence of toxins on cladocerans, as they are generally the dominant zooplankton in freshwaters, contributing up to 80% of the secondary production [33]. Furthermore, they are an important component of aquatic food webs, acting as major consumers of primary production and as a major food source for fish and invertebrate predators [33]. Species of *Daphnia* tend to be the dominant caldocerans and are model organisms for studying a range of issues [36]–[38]. Specifically, to fully understand how external factors affect *Daphnia*, many studies carefully resolve their influence on a range of distinct life history traits [26], [35], [39].

Here, therefore, we have focused on the combined effects of ammonia and microcystin, two of the main toxins that arise as end products of *Microcystis* blooms, on the life-history traits of a model cladoceran. Our research employs the model species *D. magna*, which has been used for a range of ecophysiological, behavioral, and developmental studies. For instance, *D. magna* has been used to reveal the unexpected toxicity of fatty acids on zooplankton [40], the chronic toxicity of NO_x_ to the crustacean [36], and the toxicity of nanoparticles on freshwater organisms [41]. Furthermore, several studies directly relate to cyanobacterial toxins have been conducted on this species, making *D. magna* an even more relevant model for our purposes: e.g. chronic toxicity of microcystin [42], and effects of microcystin-LR on biotransformation and oxidative stress [43].

Specifically, to obtain well-controlled and repeatable results that will allow further exploration, we chose a reliable clone of *D. magna* that has been maintained in laboratory for more than 10 years. We hypothesized that most life-history traits of *D. magna* will not only be adversely affected by ammonia and microcystin independently, but interactive effects between the two toxins will occur. Surprisingly, our data reveal that interactive effects occurred, but these were both synergistic and antagonistic, depending on the life-history traits. Our work thus stimulates a new avenue for toxicological studies, specifically on cladocera and in general on freshwater zooplankton.

## Materials and Methods

### Test organism

A laboratory clone of *Daphnia magna*, maintained for >10 years, was cultured in 200 mL beakers and fed *Scenedesmus obliquus*, at 25°C, under fluorescent light at 40 µmol photons m^−2^ s^−1^, with a light-dark period of 12∶12 h. All experiments were conducted under these conditions.

### Experimental design

New-born (<24 h-old) *D. magna*, taken from a single mother (F_0_) in a stock culture, were isolated and grown individually in 50 mL beakers and fed daily on *S. obliquus* (5.0×10^4^ cells mL^−1^). Experimental lines (F_1_, F_2_) were conditioned for maternal effects under constant conditions for two generations prior to experiments, with each new generation arising from randomly chosen individuals from the third clutch of the previous generation.

Experimental animals (F_3_) were placed randomly in 50-mL beakers. Each beaker contained one experimental animal to avoid density effects [44], and each treatment was replicated (n = 4). Ammonia test solutions were prepared by dissolving ammonium chloride (NH_4_Cl) in de-chlorinated tap water. NH_3_-N concentrations were calculated using the general equation of bases [45]:
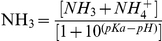
where, the calculation of pKa is based on the equation [45]: pK_a_ = 0.09018+2729.92/T, (T is in °K), pH was stable at 7.5. Based on the toxin levels in the field during degradation of heavy *Microcystis* blooms, un-ionized ammonia nitrogen concentrations (NH_3_-N) and purified microcystin-LR (MC-LR) concentrations (Express, Beijing, China) treatments were: 0, 0.366, 0.581 mg L^−1^ and 0, 10, 30, 100 µg L^−1^, respectively. Experiments followed a fully-factorial design; i.e. there were 12 treatment combinations. To maintain constant ammonia and microcystin concentrations, test solutions were replaced every two days (preliminary data indicated this maintained stable levels). Experimental conditions were identical to those described above for culturing, and *S. obliquus* concentration was maintained at 5.0×10^4^ cells mL^−1^.

 The animals were exposed to the test solutions for 14 days. During the 14-day exposure, the following parameters were recorded. The survival and moults (i.e. shed carapaces) were monitored daily. Body length was measured from above the eye to the base of the tail spine daily. Offspring production was measured daily; once counted these were removed. Also recorded were: the time to first batch of eggs appearing in the brood pouch; the time to first clutch; the size at first batch of eggs; the size at first clutch; the number of clutches per female; the number of offspring in each clutch; and the total number of offspring per female.

### Statistical analysis

Treatment effects were assessed by two-way ANOVA followed by Duncan's multiple range test (α = 0.05). Data are presented as means ± 1 SE, with different letter to indicate lack of significant differences.

## Results

### Survival and moults

**Figure 1:**
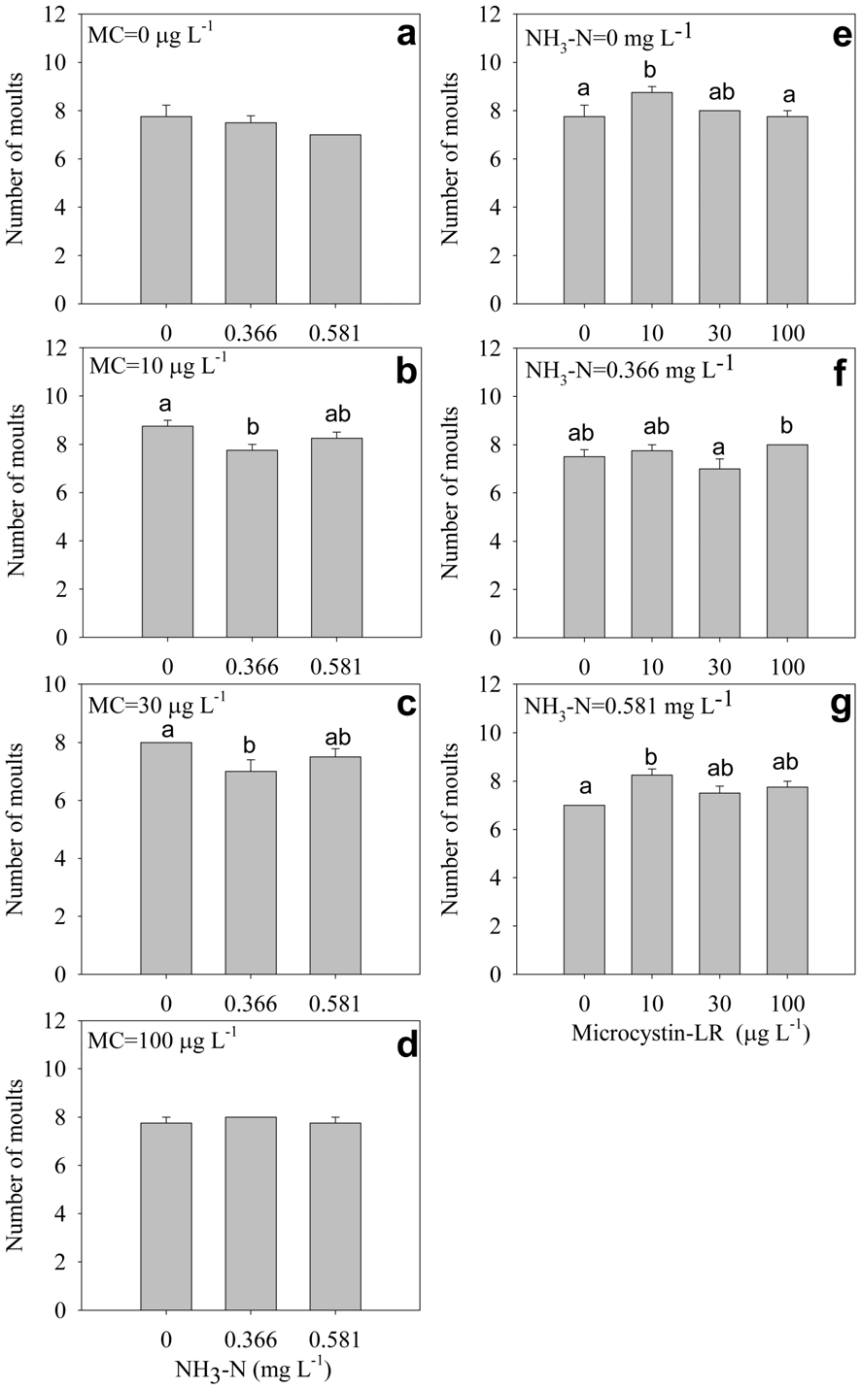
Number of moults. Effect of ammonia under different microcystin-LR concentrations (a–d) and the effect of microcystin-LR under different ammonia concentrations (e–g) on number of moults. Error bars indicate 1 SE (some error bars are too small and covered), and different letters denote significant difference at *P*<0.05.

Experimental animals in all treatments survived during the exposure and the recovery period. Two-way ANOVA showed both ammonia and microcystin had significant effects on number of moults, but their effects were independent, i.e. no interactive effect of ammonia and microcystin on number of moults (Table 1). Generally, number of moults decreased with increasing ammonia concentration under mid-dose microcystin conditions (Figure 1b–c), whereas the presence of microcystin increased number of moults under any ammonia concentrations, even significant effects were detected in some microcystin concentrations (Figure 1e–g).

**Table 1:** Results from two-way ANOVA for the factors ammonia (NH_3_-N) and microcystin-LR (MC-LR) for each response variable.

Traits	Source of variation	DF	SS	MS	F	P
Number of moults	NH_3_-N	2	2.375	1.188	4.071	0.025
	MC-LR	3	5.167	1.722	5.905	0.002
	NH_3_-N×MC	6	2.958	0.493	1.690	0.152
Time to first eggs	NH_3_-N	2	30.466	15.233	131.430	<0.001
	MC-LR	3	21.527	7.176	61.912	<0.001
	NH_3_-N×MC	6	5.797	0.966	8.336	<0.001
Time to first clutch	NH_3_-N	2	26.073	13.036	49.728	<0.001
	MC-LR	3	5.807	1.936	7.384	<0.001
	NH_3_-N×MC	6	11.552	1.925	7.344	<0.001
Size at first eggs	NH_3_-N	2	111190.912	55595.456	6.831	0.003
	MC-LR	3	170623.804	56874.601	6.988	<0.001
	NH_3_-N×MC	6	113802.785	18967.131	2.331	0.053
Size at first clutch	NH_3_-N	2	45917.540	22958.770	1.721	0.193
	MC-LR	3	84585.525	28195.175	2.114	0.116
	NH_3_-N×MC	6	303777.888	50629.648	3.795	0.005
Number of clutches	NH_3_-N	2	4.625	2.313	16.650	<0.001
	MC-LR	3	2.167	0.722	5.200	0.004
	NH_3_-N×MC	6	3.208	0.535	3.850	0.005
Number of offspring per clutch	NH_3_-N	2	64.823	32.411	9.328	<0.001
	MC-LR	3	24.863	8.288	2.385	0.085
	NH_3_-N×MC	6	33.094	5.516	1.587	0.179
Total offspring per female	NH_3_-N	2	1600.167	800.083	26.956	<0.001
	MC-LR	3	505.750	168.583	5.680	0.003
	NH_3_-N×MC	6	669.500	111.583	3.759	0.005

### Age and size at first reproduction

**Figure 2:**
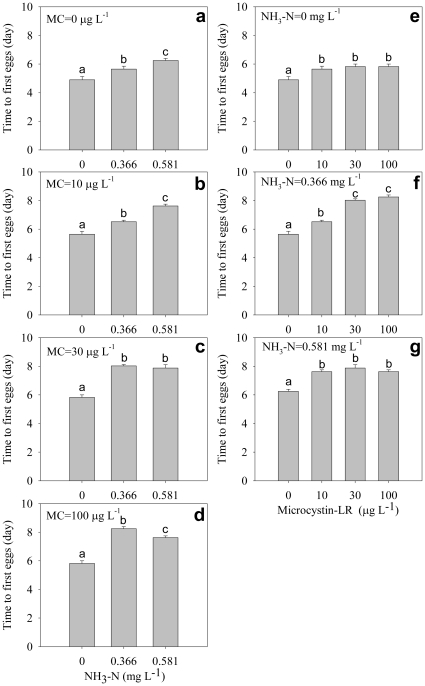
Time to first eggs. Effect of ammonia under different microcystin-LR concentrations (a–d) and the effect of microcystin-LR under different ammonia concentrations (e–g) on time to first eggs. Error bars indicate 1 SE, and different letters denote significant difference at *P*<0.05.

**Figure 3:**
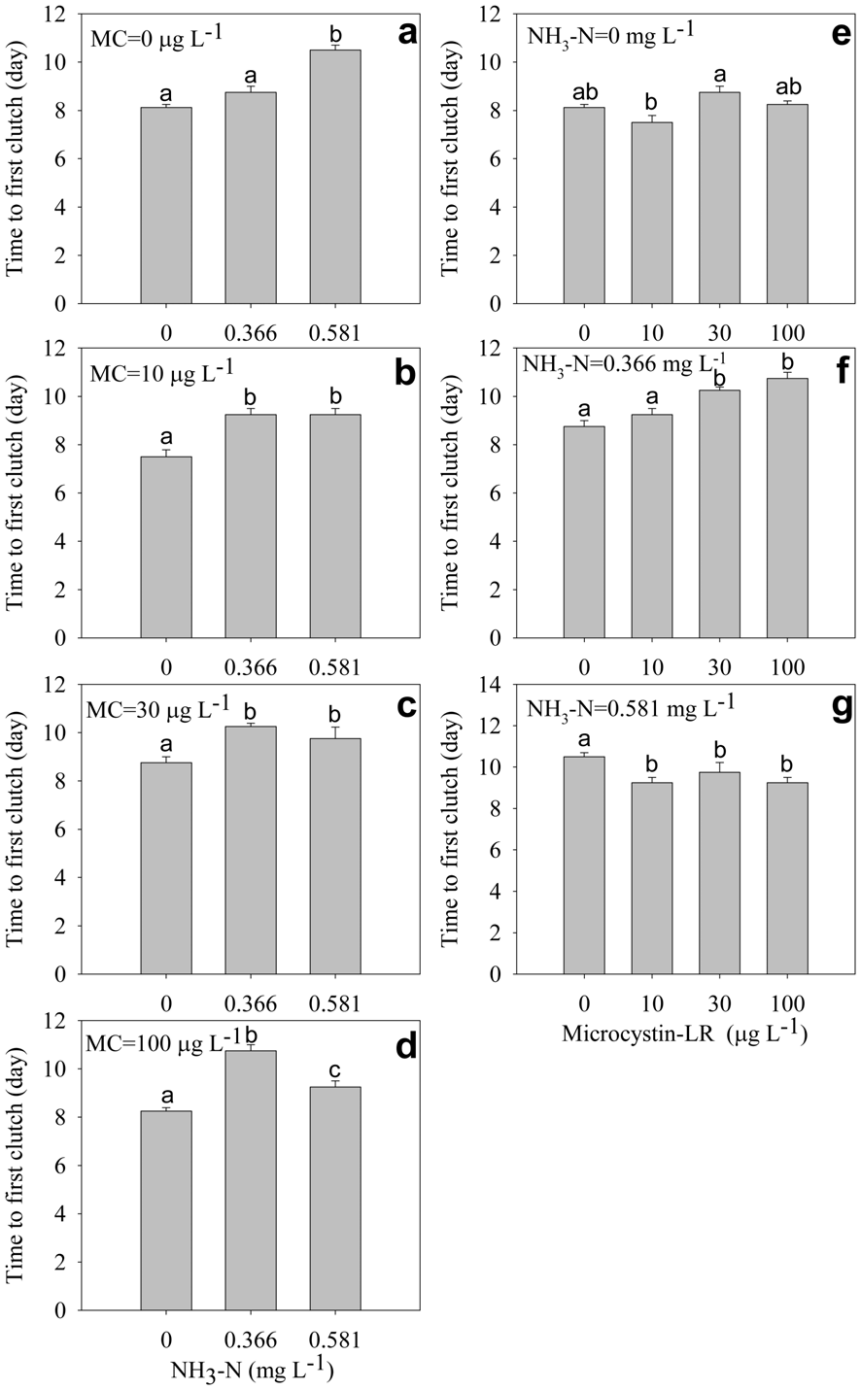
Time to first clutch. Effect of ammonia under different microcystin-LR concentrations (a–d) and the effect of microcystin-LR under different ammonia concentrations (e–g) on time to first clutch. Error bars indicate 1 SE, and different letters denote significant difference at *P*<0.05.

Both ammonia and microcystin significantly delayed time to first eggs and time to first clutch, and there was significant interaction between ammonia and microcystin on time to first eggs and first clutch (Table 1). Generally, time to first eggs was delayed with increasing ammonia concentration under any microcystin concentrations (Figure 2a–d); it was also delayed with increasing microcystin concentration under any ammonia concentrations (Figure 2e–g). The time to first clutch was delayed with increasing ammonia concentration under all microcystin concentrations (Figure 3a–d). Interestingly, under high ammonia concentrations, the presence of microcystin significantly alleviated the delayed effects of ammonia on time to first clutch (Figure 3g), although time to first clutch was delayed by microcystin under lower or no ammonia conditions (Figure 3e–f).

**Figure 4:**
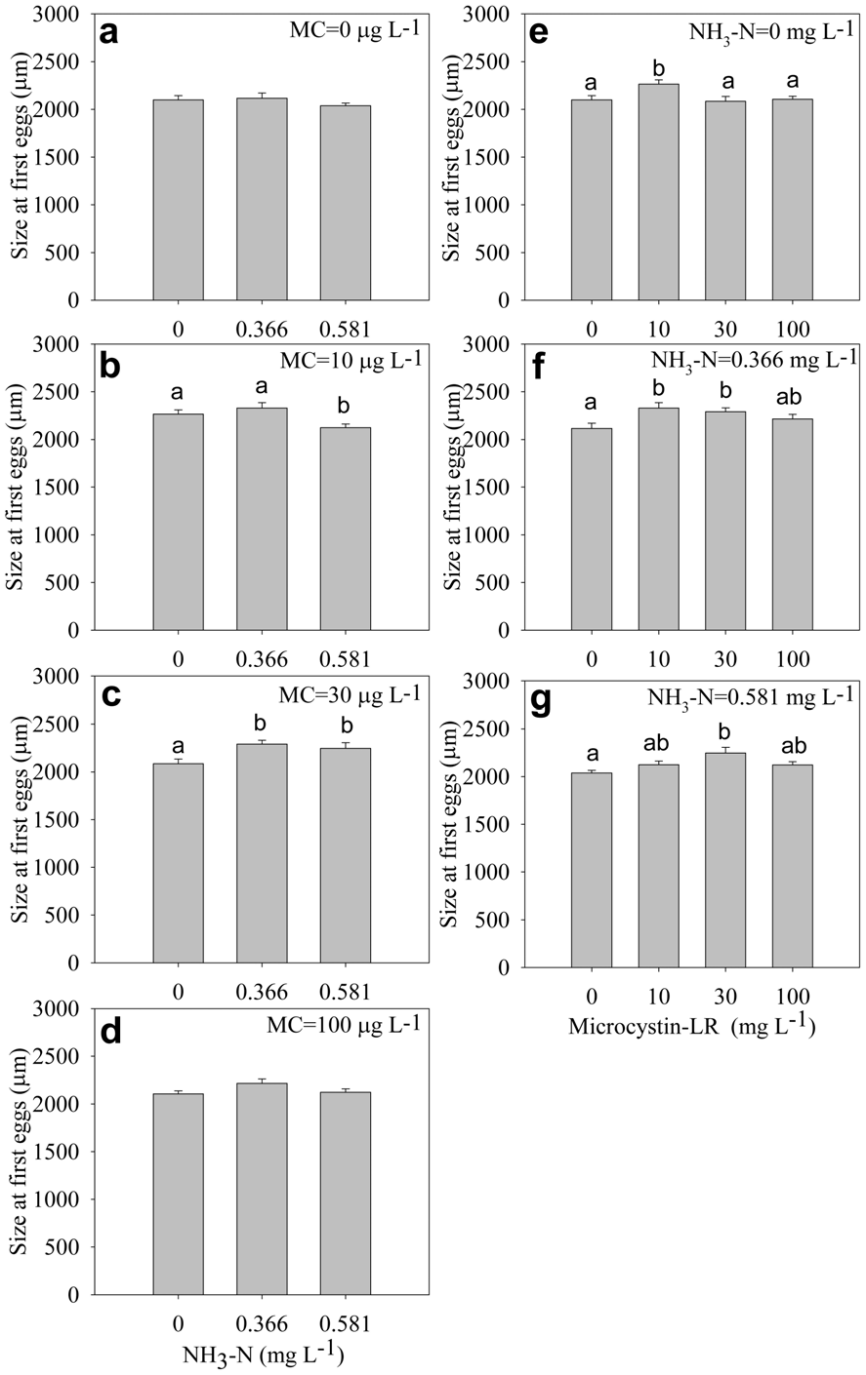
Size at first eggs. Effect of ammonia under different microcystin-LR concentrations (a–d) and the effect of microcystin-LR under different ammonia concentrations (e–g) on size at first eggs. Error bars indicate 1 SE, and different letters denote significant difference at *P*<0.05.

**Figure 5:**
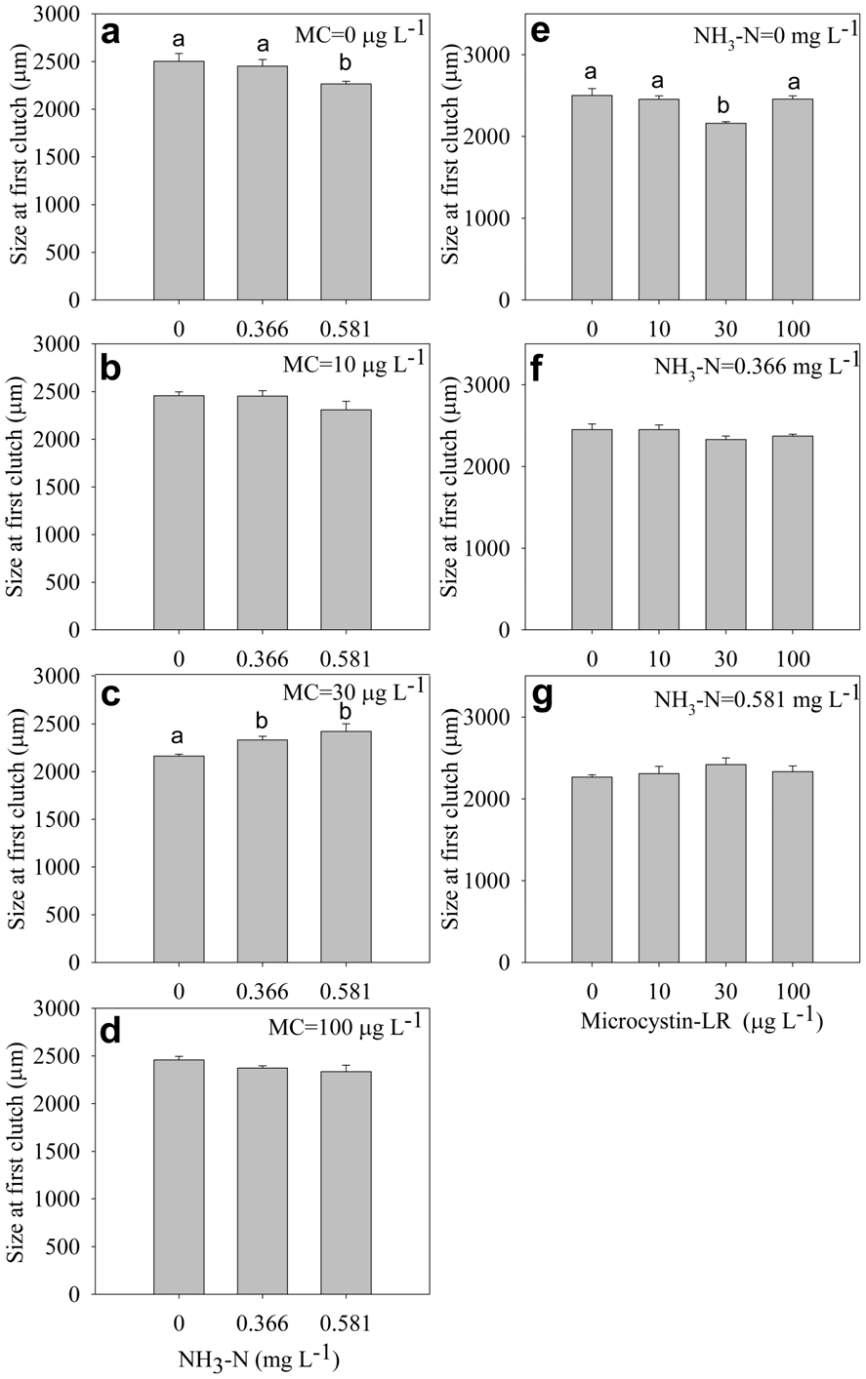
Size at first clutch. Effect of ammonia under different microcystin-LR concentrations (a–d) and the effect of microcystin-LR under different ammonia concentrations (e–g) on size at first clutch. Error bars indicate 1 SE, and different letters denote significant difference at *P*<0.05.

The size at first eggs was significantly affected by ammonia and microcystin (Table 1). In general, size at first eggs increased with increasing microycstin concentration (Figure 4e–g), but the effect of ammonia on size at first eggs was irregular (Figure 4a–d). Surprisingly, size at first clutch significantly decreased under high ammonia and high microcystin (Figure 5a,e) but remained unchanged under mixed toxins with high concentrations (Figure 5d,g), indicating significant interactive effect of ammonia and microcystin on size at first clutch (Table 1).

### Clutches and offspring

**Figure 6:**
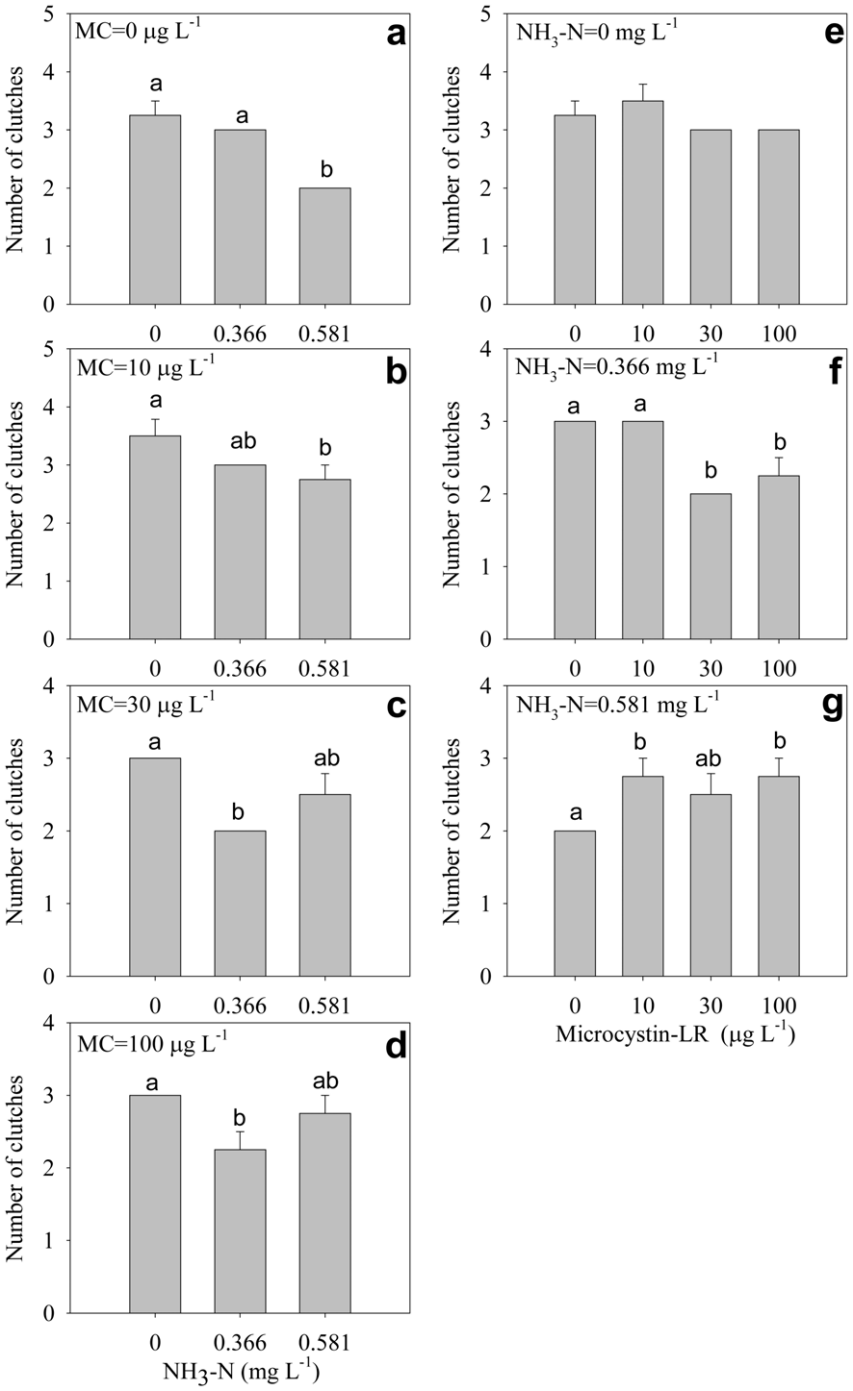
Number of clutches. Effect of ammonia under different microcystin-LR concentrations (a–d) and the effect of microcystin-LR under different ammonia concentrations (e–g) on number of clutches. Error bars indicate 1 SE (some error bars are too small and covered), and different letters denote significant difference at *P*<0.05.

Number of clutches decreased significantly with increasing ammonia concentration under all microcystin concentrations (Figure 6a–d), but the effects of microcystin on number of clutches under different ammonia concentrations were complex and seemed irregular: number of clutches was not significantly influenced by microcystin without ammonia (Figure 6e); under mid-dose ammonia, the number of clutches decreased significantly with increasing microcystin concentration (Figure 6f); under high-dose ammonia, the number of clutches increased significantly with increasing microcystin concentration (Figure 6g). There was significant interactive effect of ammonia and microcystin on the number of clutches (Table 1).

**Figure 7:**
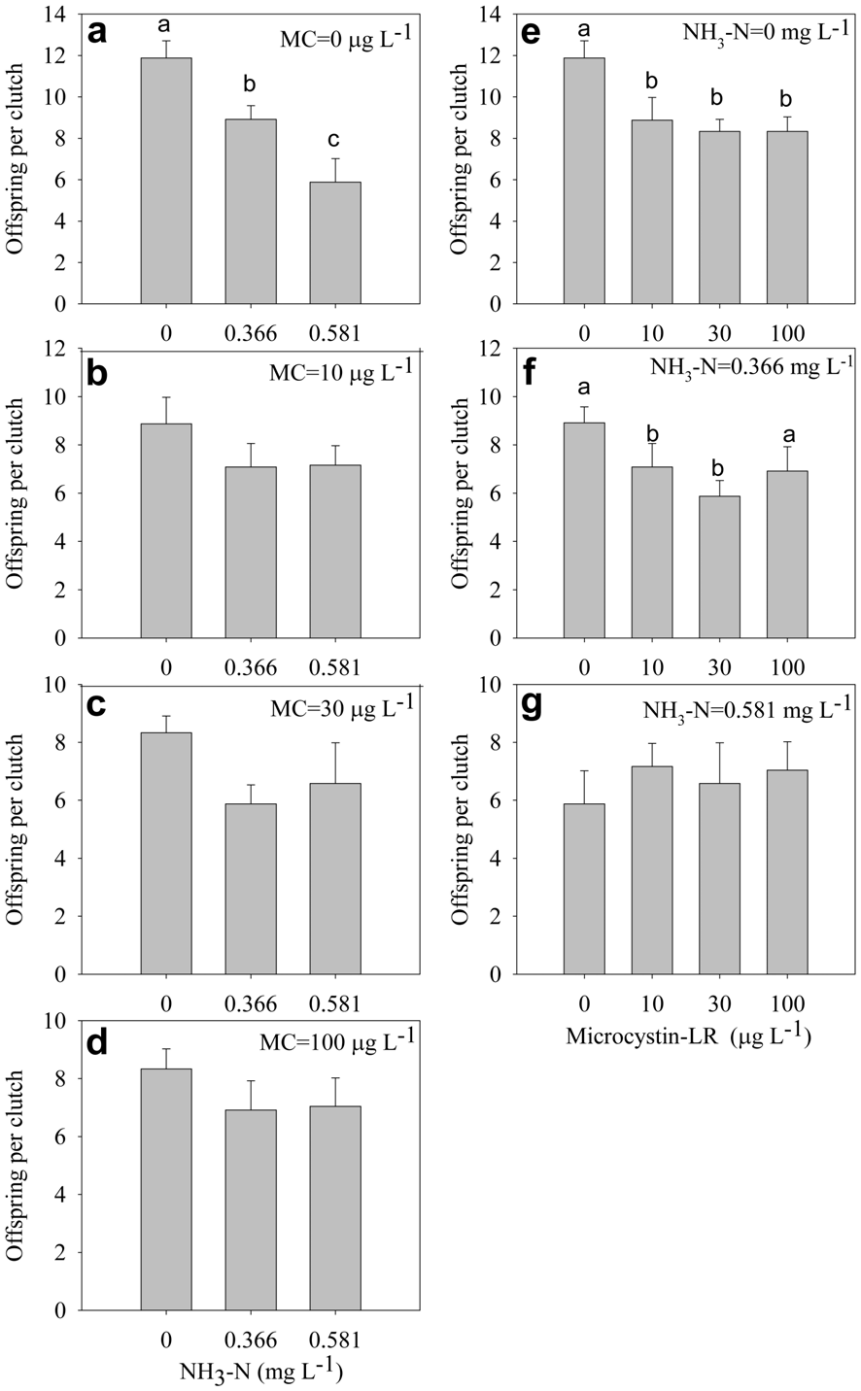
Number of offspring per clutch. Effect of ammonia under different microcystin-LR concentrations (a–d) and the effect of microcystin-LR under different ammonia concentrations (e–g) on number of offspring per clutch. Error bars indicate 1 SE, and different letters denote significant difference at *P*<0.05.

The number of offspring per clutch decreased significantly with increasing ammonia concentration without microcystin (Figure 7a), but no significant difference occurred among different ammonia concentrations under any microcystin concentrations (Figure 7b–d). Under lower ammonia concentrations, microcystin significantly decreased the number of offspring per clutch (Figure 7e, f), whereas under high ammonia concentrations, no significant difference occurred among different microcystin concentrations (Figure 7g).

**Figure 8:**
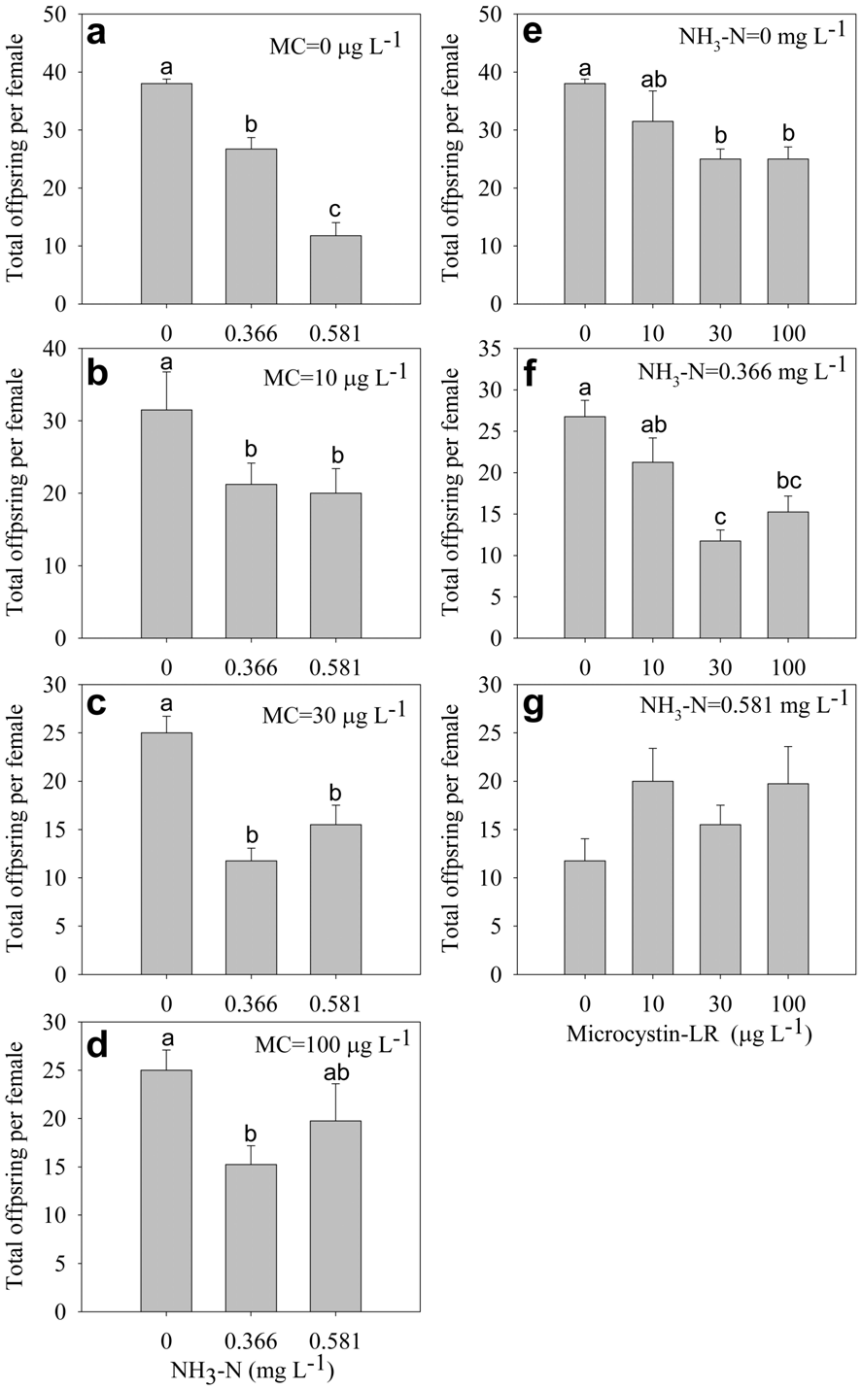
Total offspring per female. Effect of ammonia under different microcystin-LR concentrations (a–d) and the effect of microcystin-LR under different ammonia concentrations (e–g) on total offspring per female. Error bars indicate 1 SE, and different letters denote significant difference at *P*<0.05.

The total offspring per female decreased significantly with increasing ammonia concentration under all microcystin concentrations (Figure 8a–d). Under lower ammonia concentrations, microcystin significantly decreased the total offspring per female (Figure 8e,f), whereas under high ammonia concentrations, no significant difference occurred among the different microcystin concentrations (Figure 8g).

## Discussion

Using the model species *Daphina magna*, we investigated the combined effects of ammonia and microcystin-LR on life-history traits. Our main finding was that both ammonia and microcystin are substantially detrimental to *D. magna* reproduction, and expected maximum levels of ammonia [28] seemed more harmful than expected maximum levels of microcystin [9], [10]. Furthermore, the interactive effects of ammonia and microcystin occurred for most of the life-history traits; i.e. of the eight traits examined, interactive effects of microcystin and ammonia occurred for five (Table 1): time to first batch of eggs appearing in the brood pouch, time to first clutch, size at first clutch, number of clutches, and total offspring per female.

We note that although there were statistically significant effects for some of the response variables, the magnitude of the effects for many of the variables at most is ∼5 to 10%; this is especially so for the developmental variables such as time to first batch of eggs appearing in the brood pouch, time to first clutch, and size at first clutch. In contrast, the reproductive variables (number of clutches and total offspring per female), which will have direct effects on population growth, changed by as much as 50% due to treatments, and interaction altered these in some cases by ∼10–20%. As indicated in the Introduction, the specifics of these changes may vary depending on the experimental organism of study, so we shall not place emphasis on details. Rather, we use these data to illustrate that in a 14-day incubation there can be subtle and dramatic influences of these toxins on zooplankton, individually and in combination. Given predictions that cyanobacterial blooms will increase in frequency and magnitude in the future [4], we might anticipate that these individual responses, which will influence exponential population growth and competitive advantages may have pronounced effects on populations and communities. Below, in more detail, we examine these toxins independently and then consider the implication of their interactive effects.

Ammonia is toxic to most aquatic organisms, including arthropods and fish, with a range of physiological consequences [46]–[48]. We indicate that sub-lethal ammonia levels are detrimental to *D. magna* reproduction, causing delayed maturity, reductions in offspring production, and affecting maternal growth and size at maturity. Such chronic consequences of ammonia toxicity on cladocerans are well understood for other species [30]–[33], [35]. Thus, we support an increasing body of literature on the problems caused by sub-lethal ammonia concentrations and maintain that its generation and accumulation must be managed to protect aquatic ecosystems. For instance, due to bacterial degradation of *Microcystis* blooms maximum total ammonia (including ionized ammonia and un-ionized ammonia) levels in lakes can be on the order of 0.2–3.4 mg L^−1^ (e.g. in certain areas of Lake Taihu at specific times) [28], which would result, according to our data, in substantially negative effect on *D. magna*.

Microcystin-LR is a hepatotoxin produced by several cyanobacteria [49] that affects both vertebrates [50] and invertebrates [18]. Microcystin induces oxidative stress and inhibits protein phosphatases, which are main mechanisms of its toxicity [51]–[53]. Furthermore, microcystin-LR reduces *Daphnia* filtration rate, inhibiting feeding [19]. However, both chronic and acute toxicity of *Microcystis* blooms and toxins on *Daphnia* are variable, and unexpected levels of tolerance to microcystins by *Daphnia* occurs [26], [42], [54], [55]. For instance, Dao et al. [56] observed reduced neonate production and shortened time to maturity for *D. magna* at 50 µg L^−1^ of microcystin-LR. We noted similar reduced effect of microcystin on offspring produced per clutch (a parameter akin to neonate production) and total offspring per female. Meanwhile, we also noted microcystin significantly delayed time to first eggs, which is inconsistent with some other studies, probably suggesting the strain we used is more sensitive than some other studied strains [39], [56]. Also, purified microcystin seems, in some cases, not to be detrimental to *Daphnia* [39], or it only does so under very high concentrations [18].

The differences in tolerance to microcystin are probably due to the long-term maintenance of some species or clones under pristine laboratory conditions (such as the clone used in this experiment), while others, more recently isolated, have become adapted to microcysin as they regularly experiences *Microcystis* blooms [39], [57]–[59]. In our experiments, the clone of *D. magna* was maintained in the laboratory for more than 10 years, and probably has either lost its tolerance to microcystin or has not developed a resistance to it through regular exposure; in either case, our data can then be used to provide a “worst-case scenario” assessment of cyanobacterial blooms. For instance, as blooms are becoming more prevalent globally, our data are especially relevant to areas where blooms are becoming a problem.

The interactive effects of ammonia and microcystin occurred for the five traits (Table 1): the time to first batch of eggs appearing in the brood pouch, time to first clutch, size at first clutch, number of clutches, and total offspring per female. Interestingly, it seems that the interactive effects of ammonia and microcystin are synergistic on some parameters and but antagonistic on some other parameters. For example, a synergistic effect was found on the time to first eggs, i.e. *D. magna* confronted simultaneously with ammonia and microcystin, delays its time to produce the first clutch of eggs, compared with those exposed to a single toxin (Figure 2). In contrast, under lower ammonia concentrations, microcystin significantly decreased total offspring per female (Figure 8e,f), whereas under high ammonia concentrations, no difference occurred among different microcystin concentrations (Figure 8g), indicating an antagonistic effect. Another antagonistic effect occurred for the time to first clutch, under high ammonia concentrations: the presence of microcystin alleviated the delayed effects of ammonia on time to first clutch (Figure 3g), which is similar to the effect of nitrite and microcystin on *Daphnia obtusa* [39]. Thus, the above phenomenon suggested that combined effects of ammonia and microcystin on different traits of life-history are complex and unintuitive. We suggest, given the importance of *Daphnia* in food webs and the growing concerns regarding cyanobacterial blooms [1], [33], that there now is a need to rigorously assess the mechanism behind these phenomenon. We also emphasize that our results on this one model taxon should stimulate similar experiments on other cladocerans and freshwater zooplankton, as differences will undoubtedly occur between genera, species, and even clones. Revealing such variation in the response to toxins may indicate the robustness of the zooplankton community to cyanobacterial blooms.

### Conclusion

We support the wide body of literature that recognizes ammonia and microcystin as toxins. Both ammonia and microcystin can adversely affect life-history traits of *D. magna*. Furthermore, interactive effects of ammonia and microcystin occurred for five traits: the time to first batch of eggs appearing in the brood pouch, time to first clutch, size at first clutch, number of clutches, and total offspring per female. Critically, it seems that the interactive effects are synergistic on some traits (e.g. time to first eggs) and but antagonistic on some other traits (e.g. total offspring per female). This study reveals complex interactions between these toxins that require careful physiological-based study to understand the underlying mechanisms. We thus conclude by recommending an interdisciplinary approach to address this issue, possibly coupling ecophysiological techniques with more classical mechanistic physiology and modern methods of gene expression [60], [61] to untangle these issues and allow us to prepare for the inevitable expansion of cyanobacterial blooms, on a global scale.
